# Progress towards the UNAIDS 90–90-90 goals by age and gender in a rural area of KwaZulu-Natal, South Africa: a household-based community cross-sectional survey

**DOI:** 10.1186/s12889-018-5208-0

**Published:** 2018-03-02

**Authors:** Helena Huerga, Gilles Van Cutsem, Jihane Ben Farhat, Adrian Puren, Malika Bouhenia, Lubbe Wiesner, Linda Dlamini, David Maman, Tom Ellman, Jean-François Etard

**Affiliations:** 1 Clinical Research, Epicentre, 8 rue Saint-Sabin, 75011 Paris, France; 2 Medical Department, Médecins Sans Frontières, Cape Town, South Africa; 3 Centre for Infectious Disease Epidemiology and Research, University of Cape Town, Cape Town, South Africa; 4 National Institute for Communicable Diseases of the NHLS, Johannesburg, South Africa; 5 Division of Clinical Pharmacology, Department of Medicine, University of Cape Town, Cape Town, South Africa; 6 Department of Health, District, Empangeni, Uthungulu, South Africa; 7 IRD UMI 233, INSERM U1175, Université de Montpellier, Unité TransVIHMI, Montpellier, France

**Keywords:** HIV, Cascade of care, Viral load, UNAIDS targets, Africa

## Abstract

**Background:**

The Joint United Nations Programme on HIV/AIDS (UNAIDS) has developed an ambitious strategy to end the AIDS epidemic. After eight years of antiretroviral therapy (ART) program we assessed progress towards the UNAIDS 90–90-90 targets in Mbongolwane and Eshowe, KwaZulu-Natal, South Africa.

**Methods:**

We conducted a cross-sectional household-based community survey using a two-stage stratified cluster probability sampling strategy. Persons aged 15–59 years were eligible. We used face-to-face interviewer-administered questionnaires to collect information on history of HIV testing and care. Rapid HIV testing was performed on site and venous blood specimens collected from HIV-positive participants for antiretroviral drug presence test, CD4 count and viral load. At the time of the survey the CD4 threshold for ART initiation was 350 cells/μL. We calculated progression towards the 90–90-90 UNAIDS targets by estimating three proportions: HIV positive individuals who knew their status (first 90), those diagnosed who were on ART (second 90), and those on ART who were virally suppressed (third 90).

**Results:**

We included 5649/6688 (84.5%) individuals. Median age was 26 years (IQR: 19–40), 62.3% were women. HIV prevalence was 25.2% (95% CI: 23.6–26.9): 30.9% (95% CI: 29.0–32.9) in women; 15.9% (95% CI: 14.0–18.0) in men. Overall progress towards the 90–90-90 targets was as follows: 76.4% (95% CI: 74.1–78.6) knew their status, 69.9% (95% CI: 67.0–72.7) of those who knew their status were on ART and 93.1% (95% CI: 91.0–94.8) of those on ART were virally suppressed. By sex, progress towards the 90–90-90 targets was: 79%–71%–93% among women; and 68%–68%–92% among men (*p*-values of women and men comparisons were < 0.001, 0.443 and 0.584 respectively). By age, progress was: 83%–75%–95% among individuals aged 30–59 years and 64%–58%–89% among those aged 15–29 years (*p*-values of age groups comparisons were < 0.001, < 0.001 and 0.011 respectively).

**Conclusions:**

In this context of high HIV prevalence, significant progress has been achieved with regards to reaching the UNAIDS 90–90-90 targets. The third 90, viral suppression in people on ART, was achieved among women and men. However, gaps persist in HIV diagnosis and ART coverage particularly in men and individuals younger than 30 years. Achieving 90–90-90 is feasible but requires additional investment to reach youth and men.

**Electronic supplementary material:**

The online version of this article (10.1186/s12889-018-5208-0) contains supplementary material, which is available to authorized users.

## Background

The Joint United Nations Programme on HIV/AIDS (UNAIDS) has developed the ambitious 90–90-90 strategy with the objective to end the AIDS epidemic by 2030 by achieving the following three targets: 90% of all people living with HIV know their status; 90% of all people diagnosed with HIV receive sustained antiretroviral therapy (ART); and 90% of all people on ART are virally suppressed (73% of all with HIV) [1].

The achievement of these targets and in general the HIV cascade of care may be different in women and men as well as in individuals belonging to different age groups [2–5]. In addition, in the current context where test and treat and follow-up of stable HIV-positive patients on ART through viral load is recommended [6], some national programs have stopped systematic measurement of CD4 counts in newly diagnosed HIV-positive patients and/or during follow-up. Data from household-based studies on the immunological status and viral load of HIV-positive individuals can be helpful to direct program activities and resources towards underserved population groups [7].

South Africa is one of the countries with the HIV highest prevalence in the world and KwaZulu-Natal (KZN) is the province most affected by the epidemic, with an HIV prevalence of 27.9% in 2012 [8]. In 2005, the KZN Department of Health (DOH) initiated an HIV program in the Mbongolwane and Eshowe Health Service Areas, uMlalazi Municipality, KZN province, which included HIV testing, HIV care and ART initiation. In 2011, Médecins Sans Frontières (MSF) started supporting this program with large-scale HIV testing, training, mentoring and clinical support in primary care clinics to improve coverage and viral suppression.

In order to better understand the HIV epidemic at local level and adapt the strategies of intervention we assessed progress towards the UNAIDS 90–90-90 targets in the overall population and by sex and age groups.

## Methods

### Design and population

We conducted a cross-sectional household-based community survey between July and October 2013.

A two-stage stratified cluster probability sampling strategy was used for the selection of households according to the 2011 Census [9]. In total, 125 clusters of 25 households each were selected from 14 administrative units called Wards. Google Earth maps from 2011 with exhaustive identification of the households were used to sample the households to be visited by choosing randomly the first household and then sequentially the closest to the first/previous one. Field staff used Global Positioning System (GPS) receivers to find the geographic coordinates of each household.

People aged 15–59 years old living in Mbongolwane and Eshowe Health Service Areas were eligible for enrolment in the study. Those who signed a written informed consent were included.

### Study setting

Mbongolwane, a rural area, and Eshowe, the main town of the municipality, account for approximately 120,000 inhabitants [9]. Decentralization of ART care to the peripheral level was implemented gradually in this area. In 2011, the KZN province embraced the notion of nurse-initiated and managed ART (NIM-ART). MSF support to the KZN Department of Health (DOH) included prevention activities such as condom distribution, voluntary medical male circumcision, community mobilisation, large-scale community-based HIV counselling and testing, implementation of point of care CD4 testing, linkage to care, and training and mentoring of health staff in facilities in support of NIM-ART. In 2013, two district hospitals and their linked 10 primary healthcare facilities were ART-initiating centres. The survey was conducted 8 years after the initiation of the HIV program in the area. At the time of the survey the CD4 threshold for ART initiation was 350 cells/μL.

### Procedures

Prior to starting the survey, we conducted community information and mobilization activities through several channels: information on radio spots, meetings with community leaders and health facilities workers, information in schools, leaflets and posters. In order to reach a maximum of eligible individuals in their houses the survey teams visited the houses from Tuesday to Sunday. Time slots from early morning to late evening were covered in different days of the week in order to maximize the possibilities of finding the eligible participants at home. Due to the importance that blood has in the Zulu culture, the survey teams made a particular effort in explaining the purpose of collecting and storing blood and the use of it. The survey teams used face-to-face interviewer-administered questionnaires to collect information at the participant’s home on socio-demographics and history of HIV testing and care (see Additional files 1, 2 and 3). Questionnaires were developed for the Demographic and Health Surveys [10] and adapted for the study. Certified lay counsellors performed rapid HIV testing on site and provided pre and post-test counselling to the participants willing to test at home. Counsellors used Determine Rapid HIV-1/2 Antibody test kit for screening, and if positive, Unigold Rapid HIV test kit for confirmation according to the South African National guidelines for HIV Counselling and Testing. The tests were standardised and validated for this use. In addition, HIV-positivity was confirmed by ELISA at the laboratory. Survey nurses collected venous blood specimens from HIV-positive participants for antiretroviral (ARV) drug presence test, CD4 count and viral load. Venous blood samples were transported every evening to Global Clinical and Viral Laboratory in Durban. CD4 count was performed using a FACSCalibur™ device from Becton, Dickinson and Company (BD) according to standard manufacturer’s instructions on samples reported as HIV positive. Two dry blood spots (DBS) samples were prepared using the venous blood samples from each participant and transported in batches to the Department of Pharmacology laboratory at Groote Schuur Hospital, University of Cape Town, for ARV drug levels. Qualitative testing for ARV drug levels was performed for the presence of nevirapine, efavirenz and lopinavir which covered all ARV regimens in use in the public sector in the area. A liquid chromatography tandem mass spectrometry assay with a limit of quantification of 0.04 μg/mL was used for all drugs. The assay was developed and validated at the Division of Clinical Pharmacology, University of Cape Town. Viral load was performed for participants on ART for more than 6 months (determined by questionnaire) at Global Clinical and Viral Laboratory in Durban using a NucliSens EasyQ HIV-1v2.0 assay from Biomerieux according to manufacturer’s instructions. The test could quantify HIV-1 RNA over the range of 20 copies to 20 million copies for 0.5 mL sample.

### Data analyses

We calculated progression towards the 90–90-90 UNAIDS targets by estimating three proportions: HIV positive individuals who knew their status (first 90), those diagnosed who were on ART (second 90), and those on ART who were virally suppressed (third 90). Viral suppression was defined as having less than 1000 copies/mL. In addition, we calculated five steps of the HIV cascade of care using the total number of HIV positive individuals as a common denominator. ‘Diagnosed’ were the individuals who knew their HIV positive status prior to the survey; ‘Linked to care’ were those who declared having sought care for their HIV infection; ‘In care’ were those who were still receiving HIV care at the time of the survey; ‘On ART’ were those who had ARV detected in blood; ‘Virally suppressed’ were those with viral load less below 1000 copies/mL. All statistical analyses were adjusted for clustering at the level of Ward and household. Descriptive analyses are presented here with 95% confidence intervals (CI). Categorical variables were compared using proportional test. Analyses were primarily performed using Stata 13 (™StataCorp, College Station, Texas, USA).

### Ethics

The protocol was approved by the University of Cape Town Human Research Ethics Committee (HREC), the Health Research Committee of the Health Research and Knowledge Management Unit of KZN Department of Health, and the Comité de Protection de Personnes de Paris in France. All participants provided written informed consent. Participants under 18 years provided assent and their parents, guardians or caregivers provided written informed consent for them.

## Results

### Survey inclusions and participants

We visited 2377 households and we included 5649 (84.5%) participants among 6688 eligible individuals. Inclusion rate was: 3518/4008 (87.8%) among women and 2131/2680 (79.5%) among men. The remaining individuals were not included due to: refusal (8.7%), not being at home (4.9%), being incapacitated (1.0%) and other reasons (0.9%). The median age of the participants was 26 years (IQR: 19–40), 62.3% were women, 83.4% lived in rural areas, 78.8% were not living with a partner, 49.7% had completed at least secondary school, 36.3% declared no occupation and 16.6% had moved their residence in the 10 years prior to the survey or were visitors (Table 1). Thirty-two per cent of the men were under 19 years, compared to 22% of the women, possibly reflecting out-migration of adult men to seek work.

**Table 1:** Participants socio-demographic characteristics

	Women (*N* = 3518)	Men (*N* = 2131)	Total (*N* = 5649)
	n (%)	n (%)	n (%)
Age groups (years)			
- 15–19	774 (22.0)	679 (31.9)	1453 (25.7)
- 20–24	623 (17.7)	436 (20.5)	1059 (18.8)
- 25–29	497 (14.1)	295 (13.8)	792 (14.0)
- 30–34	306 (8.7)	180 (8.5)	486 (8.6)
- 35–39	283 (8.0)	134 (6.3)	417 (7.4)
- 40–44	251 (7.1)	117 (5.5)	368 (6.5)
- 45–49	259 (7.4)	93 (4.4)	352 (6.2)
- 50–54	282 (8.0)	101 (4.7)	383 (6.8)
- 55–59	243 (6.9)	96 (4.5)	339 (6.0)
Marital Status^a^			
- Never Married	2448 (69.6)	178 (83.9)	4234 (75.0)
- Married/Living Together	905 (25.8)	294 (13.8)	1199 (21.2)
- Divorced/Separated	65 (1.9)	39 (1.8)	104 (1.8)
- Widowed	97 (2.8)	10 (0.5)	107 (1.9)
Education^b^			
- No schooling	319 (9.1)	112 (5.3)	431 (7.6)
- Primary	1448 (41.2)	963 (45.2)	2411 (42.7)
- Secondary	1625 (46.2)	988 (46.4)	2613 (46.3)
- Tertiary	126 (3.6)	67 (3.2)	193 (3.4)
Place residence			
- Urban	246 (7.0)	143 (6.7)	389 (6.9)
- Semi urban	253 (7.2)	186 (8.7)	439 (7.8)
- Rural	2969 (84.4)	1742 (81.8)	4711 (83.4)
- Farm	50 (1.4)	60 (2.8)	110 (2.0)
Occupation			
- Employed	671 (19.1)	567 (26.6)	1238 (21.9)
- Unemployed	1418 (40.3)	631 (29.6)	2049 (36.3)
- Housewife/husband	439 (12.5)	26 (1.2)	465 (8.2)
- Student	876 (24.9)	756 (35.5)	1632 (28.9)
- Other	114 (3.2)	151 (7.1)	265 (4.7)
Mobility			
- Did not move	2957 (84.1)	1755 (82.4)	4712 (83.4)
- Moved residence or visitor	561 (16.0)	376 (17.6)	937 (16.6)

^a^Information on marital status missing for 3 women and 2 men

^b^Information on education missing for 1 man

### Reproductive health in women

In total, 2548 (72.4%) women had ever given birth. The median number of children per women was 2 (IQR: 1–4). At the time of the survey, 134 (3.8%) of the women were pregnant and 308 (8.8%) were breastfeeding. Of the 1259 women who had delivered in the 5 years prior to the survey (2008–2013), 1214 (96.4%) had had at least one medical antenatal care (ANC) consultation, and 920 (73.1%) had had 3 or more ANC consultations. The median number of ANC consultations was 6 (IQR: 5–7). The median month of pregnancy at the first ANC consultation was 4 months (IQR: 3–5). Out of the 799 women who had delivered in the 2 years prior to the survey, 745 (93.2%) had had an HIV test as part of their ANC.

### HIV-positive individuals

**Fig. 1:**
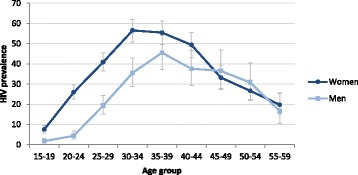
HIV prevalence by age and sex

In total, 1423 participants were HIV positive. The overall prevalence was 25.2% (95% CI: 23.6–26.9). Prevalence in women was higher than in men: 30.9% (95% CI: 29.0–32.9) vs 15.9% (95% CI: 14.0–18.0). Peak prevalence was 56.5% (95% CI: 50.9–62.0) in women at age 30–34 years and 45.5% (95% CI 37.3–54.0) in men at age 35–39 years (Fig. 1). Prevalence for age 15–29 years crudely averaged 22.3% (95% CI: 20.5–24.3) in women and 6.2% (95% CI: 5.1–7.6) in men, increasing dramatically from age 15 to 29 (3.9% to 55.0% in women and 1.5% to 26.7% in men). HIV positive mothers who had delivered in the 5 years prior to the study had a higher proportion of children who had died than HIV negative mothers: 5.3% vs 2.5% (*p* = 0.010). Of the 1400 HIV-positive participants with a CD4 count, 130 (9.3%) had a CD4 count below 200 cells/μL, 255 (18.2%) between 200 and 349 cells/μL, 363 (25.9%) between 350 and 499 cells/μL and 652 (46.6%) over or equal to 500 cells/μL. Median CD4 count was 483 cells/μL (IQR: 332–665). Among the 655 individuals not on ART, 78 (11.9%) had a CD4 count below 200 cells/μL and 138 (21.1%) between 200 and 349 cells/μL. Among the 741 individuals on ART, 52 (7.0%) had a CD4 count below 200 cells/μL and 115 (15.5%) between 200 and 349 cells/μL. Of the participants with viral load below 1000 copies/mL, 5.7% (95% CI: 4.2–7.5) had a CD4 below 200 cells/μL and 19.7% (95% CI: 17.2–22.6) a CD4 below 350 cells/μL (Table 2).

**Table 2:** CD4 count in HIV-positive individuals with viral load below 1000 copies/mL according to time on ART

	Not on ART (*N* = 106) n (%)	ART< 6 m (*N* = 149) n (%)	ART ≥6 m & < 12 m (*N* = 73) n (%)	ART ≥12 & < 24 m (*N* = 97) n (%)	ART≥24 m (*N* = 370) n (%)	All (*N* = 795) n (%)
< 200	3 (2.8)	16 (10.7)	5 (6.8)	5 (5.2)	16 (4.3)	45 (5.7)
200–349	8 (7.6)	23 (15.4)	20 (27.4)	15 (15.5)	46 (12.4)	112 (14.1)
350–499	26 (24.5)	42 (28.2)	22 (30.1)	31 (32.0)	77 (20.8)	198 (24.9)
≥500	69 (65.1)	68 (45.6)	26 (35.6)	46 (47.4)	231 62.4)	440 (55.4)

### Progress towards 90–90-90 targets

**Fig. 2:**
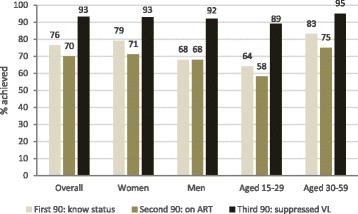
Progress towards 90–90-90 UNAIDS targets by sex and age group. First 90: proportion of HIV-positive individuals who know their status; Second 90: proportion of individuals on ART among those who know their status; Third 90: proportion of individuals with suppressed viral load among those on ART

UNAIDS targets were partially achieved with 76.4% (95% CI: 74.1–78.6) of all HIV-positive who knew their status, 69.9% (95% CI: 67.0–72.7) of them being on ART, and 93.1% (95% CI: 91.0–94.8) of the people treated being virally suppressed (Fig. 2). Progress towards the first target differed by sex and age. HIV diagnosis was: 79.0% (95% CI: 76.4–81.4) in women versus 68.3% (95% CI: 63.1–73.1) in men (*p* < 0.001); and 83.3% (95% CI: 80.6–85.7) in individuals aged 30–59 years versus 64.0% (95% CI: 59.9–67.9) in those aged 15–29 years (*p* < 0.001). Progress towards the second and third targets differed by age but not by sex. ART among individuals diagnosed was: 70.5% (95% CI: 67.7–73.2) in women versus 67.9% (95% CI: 60.8–74.2) in men (*p* = 0.443); and 75.1% (95% CI: 71.5–78.4) in individuals aged 30–59 years versus 57.6% (95% CI: 51.8–63.2) in those aged 15–29 years (*p* < 0.001). Viral suppression in individuals on ART was: 93.4% (95% CI: 91.1–95.1) in women versus 92.1% (95% CI: 86.0–95.7) in men (*p* = 0.584); and 94.5% (95% CI: 92.3–96.0) in individuals aged 30–59 years versus 89.0% (95% CI: 82.7–93.1) in those aged 15–29 years (*p* = 0.011).

**Fig. 3:**
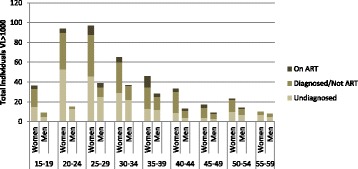
HIV positive participants with viral load ≥1000 copies/mL by gender and age group according to their diagnosis and treatment status

We also looked at other components of the cascade of care and the proportions of ART and viral suppression among all HIV-positive individuals. Of the 1384 HIV-positive participants with complete information on HIV diagnosis, ARV presence in blood and viral load, 71.0% (95% CI: 68.6–73.4) were linked to care, 62.7% (95% CI: 59.8–65.6) were in care, 53.5% (95% CI: 50.6–56.3) were on ART, 57.4% (95% CI: 54.6–60.1) were virally suppressed. The largest gaps in the cascade of care occurred in men and people aged 15–29 years (Table 3). Regarding viral suppression, 60.3% (95% CI: 57.4–63.1) of all HIV-positive women were virally suppressed compared to 47.9% (95% CI: 41.7–54.1) of the HIV-positive men and 66.2% (95% CI: 63.0–69.2) of the HIV-positive individuals aged 30–59 years were virally suppressed compared to 41.3% (95% CI: 36.9–45.9) of those aged 15–29 years. Of the 590 HIV-positive participants virally unsuppressed, 279 (47.3%) were undiagnosed and 260 (44.1%) were diagnosed but not on ART. A breakdown by age and sex is given in Fig. 3.

**Table 3:** Steps of the HIV cascade of care by sex and age groups - proportions among all HIV-positive individuals

	Women (*N* = 1056)		Men (*N* = 328)		Age 15–29 years (*N* = 491)		Age 30–59 years (*N* = 893)		Total (*N* = 1384)	
	%	95% CI	%	95% CI	%	95% CI	%	95% CI	%	95% CI
Diagnosed	79.0	76.4–81.4	68.3	63.1–73.1	64.0	59.9–67.9	83.3	80.6–85.7	76.4	74.1–78.6
Linked to care	74.3	71.6–76.9	60.4	54.2–66.1	57.2	53.0–61.3	78.6	75.6–81.3	71.0	68.6–73.4
In care	66.5	63.5–69.3	50.6	44.6–56.6	46.6	42.2–51.2	71.6	68.3–74.6	62.7	59.8–65.6
On ART	55.7	52.8–58.5	46.3	40.5–52.3	36.9	32.6–41.3	62.6	58.9–66.1	53.5	50.6–56.3
Viral load < 1000	60.3	57.4–63.1	47.9	41.7–54.1	41.3	36.9–45.9	66.2	63.0–69.2	57.4	54.6–60.1

## Discussion

In this area of KwaZulu-Natal, eight years into the public ART program, of which two with MSF support, we found that significant but insufficient progress towards the 90–90-90 UNAIDS targets was achieved. Progress towards the first and second targets was moderate and was particularly poor in men and individuals aged 15–29 years. The third target was achieved (or very close to achievement) in all sex and age categories. This progress has been made in a context of high HIV prevalence where one quarter of the overall population is HIV positive.

These findings suggest that achieving the UNAIDS 2020 targets of 90–90-90 is feasible in South Africa, but will require additional community-based investments in testing and ART initiation especially among young people and men. Investments to reach men may need to include strategies to improve HIV knowledge [11]. A household-based survey conducted in the 2 years following ours in Botswana has reported a high coverage: 83.3% of individuals knew their status, 87.4% of those were on ART, and 96.5% of those on ART had a viral load of 400 copies/mL or less (70.2% of all people with HIV) in a context of high HIV prevalence, 29% [4]. The early initiation and strong political leadership of the ART programme in Botswana might partially explain the relatively high ART coverage achieved at a time when both South African and Botswana guidelines recommended a CD4 threshold for ART initiation of 350 cells/μL. However, a household-based survey conducted the year following ours in another area of KwaZulu-Natal, found lower rates of HIV-positivity awareness (65% of the HIV-positive women and 52% of the men), similar rates of ART among those who knew their status (70% in women and 69% in men) and lower rates of viral suppression (90% in women and 85% in men) compared to our findings [3]. Other studies in KZN have shown lower proportions of ART coverage among HIV-positive individuals than ours [12–14] and at national level only 33% of the HIV-positive individuals are on ART and 24% are virally suppressed [15]. Similarly to others in this context [13, 16–19], in the area surveyed the largest losses in the HIV cascade of care occurred on diagnosis and on linkage from diagnosis into ART care. In addition, the cascade in men and people 15–29 years of age showed greater falls at each step [20, 21].

High incidence in the past associated with increased access to ART [22–25], and other factors [26] may explain the current picture of a very high prevalence. Prevalence in women increased dramatically from 15 years with a peak at 30–34 years. A rapid increase (though lower) was also observed in men but with a lag of around 5 years of age. Similar prevalence in women and men after 45 years of age could be a reflection of a differential mortality by age groups in the pre-ART era, a higher HIV incidence at older ages in men compared to women, or other competing risks such as maternal mortality [24, 27–29]. The 2012 national survey found similar age/gender trends at national level [8]. Regarding the immunological status of the people living with HIV, although the proportion in an advanced stage of HIV disease with CD4 below 200 cells/μL was relatively low, the fact that more than half were not on ART highlights that a non-negligible proportion of people with HIV don’t access care or access it very late, with significant risk of morbidity and mortality. These findings support current recommendations that HIV programmes retain the capacity to perform CD4 cell count at baseline and in case of treatment failure, as this remains one of the best predictors of general patient wellness, disease progression and mortality risk [30]. They also support the need for innovative strategies to reach individuals with high barriers to HIV testing before they develop advanced disease, such as self-testing and home-based testing.

Our study has some limitations. Some information, such as HIV status awareness used in the cascade of care for the identification of individuals already diagnosed and linked to care, was self-reported, which may have led to misclassifications. Otherwise, most of the results, crucially including ART coverage, are based on laboratory data.

## Conclusions

Significant progress has been achieved in this area with regards to reaching the UNAIDS 90–90-90 targets. The third 90, viral suppression in people on ART, was achieved among women and men. However, further efforts on diagnosis and ART initiation are needed in order to reach the first and second targets particularly in men and individuals younger than 30 years. Indeed, almost half of the people virally unsuppressed were undiagnosed. Achieving 90–90-90 is feasible but requires significant additional investment.

## Additional files

Additional file 1: Mbongolwane survey Household questionnaire: questions to the head of the household. (PDF 32 kb)

Additional file 2: Mbongolwane survey Women questionnaire: questions to the individual female participants. (PDF 63 kb)

Additional file 3: Mbongolwane survey Men questionnaire: questions to the individual male participants. (PDF 54 kb)

## Abbreviations

ANC: antenatal care
ART: antiretroviral therapy
ARV: antiretroviral
DBS: dry blood spots
DOH: Department of Health
GPS: Global Positioning System
HREC: Human Research Ethics Committee
KZN: KwaZulu-Natal
MSF: Médecins Sans Frontières
NIM-ART: nurse-initiated and managed ART
UNAIDS: United Nations Programme on HIV/AIDS
