# Supplementary material for: Progress towards the UNAIDS 90–90-90 goals by age and gender in a rural area of KwaZulu-Natal, South Africa: a household-based community cross-sectional survey
Source: BMC Public Health. 2018 Mar 2;18:303. doi: 10.1186/s12889-018-5208-0 (PMC5833029; doi:10.1186/s12889-018-5208-0)
Supplement: Supplementary file 1 — Mbongolwane survey Household questionnaire: questions to the head of the household. (PDF 32 kb) [file 12889_2018_5208_MOESM1_ESM.pdf]

MBONGOLWANE & ESHOWE HIV IMPACT IN POPULATION SURVEY - HOUSEHOLD QUESTIONNAIRE
SOUTH AFRICA - KWAZULU NATAL - UMLALAZI DISTRICT
EPICENTRE - MEDECINS SANS FRONTIERES

IDENTIFICATION (1)

WARD ______

IZIGODI ______

VILLAGE / SETTLEMENT / FARM ______

NAME OF HOUSEHOLD HEAD ______

PHONE NUMBER ______

CLUSTER NUMBER . . . .

HOUSEHOLD NUMBER . . . .

INTERVIEWER VISITS

	1	2	3	FINAL VISIT
DATE	______	______	______	DAY<br>MONTH<br>YEAR
INTERVIEWER'S NAME	______	______	______	INT. ID
RESULT*	______	______	______	RESULT*
NEXT VISIT: DATE<br>TIME	______<br>______	______<br>______		TOTAL NUMBER OF VISITS

*RESULT CODES:

1 COMPLETED
2 NO HOUSEHOLD MEMBER AT HOME OR NO COMPETENT RESPONDENT AT HOME AT TIME OF VISIT
3 ENTIRE HOUSEHOLD ABSENT FOR EXTENDED PERIOD OF TIME
4 POSTPONED
5 REFUSED
6 DWELLING VACANT OR ADDRESS NOT A DWELLING
7 DWELLING DESTROYED
8 DWELLING NOT FOUND
98 OTHER ______
(SPECIFY)

TOTAL PERSONS IN HOUSEHOLD

TOTAL ELIGIBLE WOMEN

TOTAL ELIGIBLE MEN

LINE NO. OF RESPONDENT TO HOUSEHOLD QUESTIONNAIRE

SUPERVISOR	OFFICE EDITOR
NAME ______	

THIS PAGE IS INTENTIONALLY BLANK

HOUSEHOLD SCHEDULE

LINE NO.	MEMBERS OF HOUSEHOLD AND VISITORS	RELATIONSHIP TO HEAD OF HH	SEX	RESIDENCE					AGE	DATE OF ARRIVAL	ELIGIBILITY	
					IF HOUSEHOLD MEMBER							
1	2	3	4	5	6	7	8	9	10	11	12	13
						IF DOES NOT LIVE 4 OR MORE NIGHTS A WEEK						
	Please give me the names of the people who belong to your household and guests of the household who stayed here last night, starting with the head of the household.<br><br>*AFTER LISTING THE NAMES AND RECORDING THE RELATIONSHIP AND SEX FOR EACH PERSON, ASK QUESTIONS 2A-2C LISTING IS COMPLETE.*<br><br>*THEN ASK APPROPRIATE QUESTIONS IN COLUMNS FOR EACH PERSON*<br><br>*IF ELIGIBLE STICK NUMBER ON THE RIGHT OF THE NAME*	What is the relationship of (NAME) to the head of the household?<br><br>*SEE CODES BELOW*	Is (NAME) male or female?	Does (NAME) belong to the household or is (NAME) a visitor?<br><br>*CHECK DEFINITION BELOW*	Did (NAME) spend 4 or more nights a week in this place last month?	How often does (NAME) come to the household?<br><br>YEAR<br>2= 1-2 TIMES A YEAR<br>3= >2 TIMES/YEAR BUT < 1 TIME/MONTH<br>4= >ONCE A MONTH	Where is located the place (NAME) live the other nights?<br>1= In Umlalazi Municip<br>2= In another municipality in KZN<br>3 = In other province than KZN<br>5= In other country than South Africa	***If located in other country***<br>Which country is that?<br><br>1=Mozambique<br>2= Zimbabwe<br>3=Swaziland<br>98=Other country; Specify	How old is (NAME)?<br><br>IF 95 OR MORE, RECORD '95'.	When did (NAME) arrive in the household?<br><br>1= <1 YEAR<br>2= BETWEEN 1 AND 3 YEARS<br>3= >3 YEARS	*CIRCLE LINE NUMBER OF ALL WOMEN AGE 15-59*	*CIRCLE LINE NUMBER OF ALL MEN AGE 15-59*
01			1 2<br>M F	1 2<br>HH V	1 2<br>Y N			______	IN YEARS		01	01
02			M F	HH V	Y N			______			02	02
03			M F	HH V	Y N			______			03	03
04			M F	HH V	Y N			______			04	04
05			M F	HH V	Y N			______			05	05
06			M F	HH V	Y N			______			06	06
07			M F	HH V	Y N			______			07	07
08			M F	HH V	Y N			______			08	08
09			M F	HH V	Y N			______			09	09
10			M F	HH V	Y N			______			10	10

CODES FOR Q. 3: RELATIONSHIP TO HEAD OF HOUSEHOLD

01 = HEAD
02 = WIFE OR HUSBAND
03 = SON OR DAUGHTER
04 = SON OR DAUGHTER -IN-LAW
05 = GRANDCHILD
06 = PARENT
07 = PARENT-IN-LAW
08 = BROTHER OR SISTER
09 = OTHER RELATIVE
10 = ADOPTED/FOSTER/STEPCHILD
11 = NOT RELATED
99 = DON'T KNOW

Household member:
Group of people who live together and provide themselves jointly with food and/or other essentials for living, or a single person who lives alone

Visitor:
Person who slept in the house the night preceding the interview but who does not belong to the household

	Names of the people who belong to the household and guests	Relationship to the head of the household?	Male or female?	Belong to household or visitor?	Live in place 4 or > nights a week?	*IF DOES NOT LIVE 4 OR MORE NIGHTS A WEEK* How often visit the household?	Where is located the place (NAME) live the other nights?	*If located in other country* Which country is that?	How old is (NAME)?	When arrive in the household?	*CIRCLE WOMEN AGE 15-59*	*CIRCLE MEN AGE 15-59*
			1 2	1 2					IN YEARS			
11			M F	HH V							11	11
12			M F	HH V	Y N						12	12
13			M F	HH V	Y N						13	13
14			M F	HH V	Y N						14	14
15			M F	HH V	Y N						15	15
16			M F	HH V	Y N						16	16
17			M F	HH V	Y N						17	17
18			M F	HH V	Y N						18	18
19			M F	HH V	Y N						19	19
20			M F	HH V	Y N						20	20

TICK HERE IF CONTINUATION SHEET USED ☐

2A) Just to make sure that I have a complete listing: are there any other people such as small children or infants that we have not listed?
YES ☐ → ADD TO TABLE NO ☐

2B) Are there any other people who may not be members of your family, such as domesticworkers, lodgers, or friends who usually live here?
YES ☐ → ADD TO TABLE NO ☐

2C) Are there any guests or temporary visitors staying here, or anyone else who stayed here last night, who have not been listed?
YES ☐ → ADD TO TABLE NO ☐

CODES FOR Q. 3: RELATIONSHIP TO HEAD OF HOUSEHOLD

01 = HEAD	07 = PARENT-IN-LAW
02 = WIFE OR HUSBAND	08 = BROTHER OR SISTER
03 = SON OR DAUGHTER	09 = OTHER RELATIVE
04 = SON OR DAUGHTER -IN-LAW	10 = ADOPTED/FOSTER/STEPCHILD
05 = GRANDCHILD	11 = NOT RELATED
06 = PARENT	99 = DON'T KNOW

HOUSEHOLD SCHEDULE (2)

LINE NO.	PAST RESIDENTS	RELATIONSHIP TO HEAD OF HOUSEHOLD	SEX	TYPE	AGE	TIME TO DEATH OR MIGRATION	REASON OF MIGRATION	DESTINATION OF	
1	2	3	4	5	6	7	8	9	10
	Please give me the names of the people who were living in your household last Christmas who left more than 2 weeks ago or have since died<br>AFTER LISTING THE NAMES AND RECORDING THE RELATIONSHIP AND SEX FOR EACH PERSON, ASK QUESTIONS 3A-3C LISTING IS COMPLETE.<br>THEN ASK APPROPRIATE QUESTIONS IN COLUMNS FOR EACH PERSON	What is the relationship of (NAME) to the head of the household?<br>SEE CODES BELOW.	Is (NAME) male or female?	Did (NAME) leave the household or die?<br>1= LEFT<br>2= DIED	How old was (NAME) when he/she (LEFT /DIED)?<br>IF 95 OR MORE, RECORD '95'.	How many months ago did (NAME) leave or die?<br>RECORD IN MONT	What is the reason (NAME) left?<br>1= WORK RELATED<br>2= FAMILY<br>3= FRIENDS<br>98= OTHER<br>If other, specify	Where did (NAME) move to?<br>1= UMLALAZI MUNICIPALITY<br>2= ANOTHER MUNICIPALITY IN KZN<br>3= OTHER PROVINCE THAN KZN<br>5= OTHER COUNTRY THAN SOUTH AFRICA	*If located in other country*<br>Which country is that?<br>1=Mozambique<br>2= Zimbabwe<br>3=Swaziland<br>98=OTHER, specify
			1 2	1 2	IN YEARS	IN MONTHS			
1			M F	L D					
2			M F	L D					
3			M F	L D					
4			M F	L D					
5			M F	L D					
6			M F	L D					
7			M F	L D					
8			M F	L D					
9			M F	L D					
10			M F	L D					

CODES FOR Q. 3: RELATIONSHIP TO HEAD OF HOUSEHOLD

01 = HEAD
02 = WIFE OR HUSBAND
03 = SON OR DAUGHTER
04 = SON-IN-LAW OR DAUGHTER-IN-LAW
05 = GRANDCHILD
06 = PARENT
07 = PARENT-IN-LAW
08 = BROTHER OR SISTER
09 = OTHER RELATIVE
10 = ADOPTED/FOSTER/STEPCHILD
11 = NOT RELATED
99 = DON'T KNOW

	Please give me the names of the people who were living in your household last Easter, who left more than 2 weeks ago or have since died	What is the relationship of (NAME) to the head of the household?	Is (NAME) male or female?	Did (NAME) leave the household or die?	How old was when he/she (LEFT /DIED)?	How many months ago did (NAME) leave or die?	What is the reason (NAME) left?	Where did (NAME) move to?	*If located in other country* Which country is that?
			1 2	1 2	IN YEARS	IN MONTHS			
11			M F	L D					
12			M F	L D					
13			M F	L D					
14			M F	L D					
15			M F	L D					
16			M F	L D					
17			M F	L D					
18			M F	L D					
19			M F	L D					
20			M F	L D					

TICK HERE IF CONTINUATION SHEET USED ☐

3A) Just to make sure that I have a complete listing: are there any other people such as small children or infants that we have not listed who have left or died?
YES ☐ → ADD TO TABLE NO ☐

3B) Are there any other people who may not be members of your family, such as domestic workers, lodgers, or friends who used to live here and have left or died?
YES ☐ → ADD TO TABLE NO ☐

3C) If no member of the household left or died, tick this box ☐

CODES FOR Q. 13: RELATIONSHIP TO HEAD OF HOUSEHOLD

01 = HEAD
02 = WIFE OR HUSBAND
03 = SON OR DAUGHTER
04 = SON-IN-LAW OR DAUGHTER-IN-LAW
05 = GRANDCHILD
06 = PARENT
07 = PARENT-IN-LAW
08 = BROTHER OR SISTER
09 = OTHER RELATIVE
10 = ADOPTED/FOSTER/ STEPCHILD
11 = NOT RELATED
99 = DON'T KNOW

06 = PARENT
07 = PARENT-IN-LAW
08 = BROTHER OR SISTER
09 = OTHER RELATIVE
10 = ADOPTED/FOSTER/ STEPCHILD
11 = NOT RELATED
99 = DON'T KNOW
