# Supplementary material for: Progress towards the UNAIDS 90–90-90 goals by age and gender in a rural area of KwaZulu-Natal, South Africa: a household-based community cross-sectional survey
Source: BMC Public Health. 2018 Mar 2;18:303. doi: 10.1186/s12889-018-5208-0 (PMC5833029; doi:10.1186/s12889-018-5208-0)
Supplement: Supplementary file 2 — Mbongolwane survey Women questionnaire: questions to the individual female participants. (PDF 63 kb) [file 12889_2018_5208_MOESM2_ESM.pdf]

MBONGOLWANE 1 ESHOWE HIV IMPACT IN POPULATION SURVEY - INDIVIDUAL QUESTIONNAIRE
SOUTH AFRICA - KWAZULU NATAL -UMLALAZI DISTRICT
EPICENTRE - MEDECINS SANS FRONTIERES

IDENTIFICATION

WARD ______________________

IZIGODI ______________________

VILLAGE / SETTLEMENT / FARM ______________________

NAME OF HOUSEHOLD HEAD ______________________

PHONE NUMBER ______________________

CLUSTER NUMBER . . . . [ ][ ][ ]

HOUSEHOLD NUMBER . . . . [ ][ ]

NAME AND LINE NUMBER OF WOMAN ______________________ [ ][ ]

IDENTIFICATION NUMBER [ ]

INTERVIEWER VISITS

	1	2	3	FINAL VISIT
DATE	________	________	________	DAY [ ][ ]<br>MONTH [ ][ ]<br>YEAR [ ][ ][ ][ ]
INTERVIEWER'S NAME	________	________	________	INT. ID [ ][ ]
RESULT*	________	________	________	RESULT* [ ]
NEXT VISIT: DATE	________	________		TOTAL NUMBER OF VISITS [ ]
TIME	________	________		

*RESULT CODES:

1 COMPLETED
2 NOT AT HOME
3 POSTPONED
4 REFUSED
5 PARTLY COMPLETED
6 INCAPACITATED
8 OTHER ______________________ (SPECIFY)

SUPERVISOR	OFFICE EDITOR
[ ]<br>NAME ________ [ ][ ]	[ ][ ]

THIS PAGE IS INTENTIONALLY BLANK

SECTION 1. RESPONDENT'S BACKGROUND

INTRODUCTION AND CONSENT

NO.	QUESTIONS AND FILTERS	CODING CATEGORIES	SKIP
101	RECORD THE TIME.	HOUR . . . . [ ][ ]<br>MINUTES . . . . [ ][ ]	
102	When were you born (month and year)?	MONTH . . . . [ ][ ]<br>DON'T KNOW MONTH . . . . 99<br>YEAR . . . . [ ][ ][ ][ ]<br>DON'T KNOW YEAR 9999	
103	How old were you at your last birthday?<br>COMPARE AND CORRECT 102 AND/OR 103 IF INCONSISTENT.	AGE IN COMPLETED YEARS [ ][ ]	
104	What is the highest level of school you attended: primary, secondary, or higher?	Incomplete Primary (< than grade 7) . 1<br>Primary - Complete grade 7 GEC . . . 2<br>Secondary - Complete Matric . . . . 3<br>Tertiary education . . . . 4<br>No schooling 5	
105	Were you born in Umlalazi Municipality?	YES . . . . 1<br>NO 2	1 → 108
106	Where were you born?	In another municipality within Kwazulu-Natal . . . . 1<br>In a province other than Kwazulu-Natal in South Africa . . . . 2<br>In a country other than South Africa . 3	1, 2 → 108
107	In which country were you born?	Mozambique . . . . 1<br>Zimbabwe . . . . 2<br>Swaziland . . . . 3<br>OTHER ______________ 98<br>(SPECIFY)	
108	Do you belong to this househould or are you a visitor?	Belong to household . . . . 1<br>Visitor 2	2 → 112
109	Did you spend 4 or more nights a week in this place in the previous month?	YES . . . . 1<br>NO 2	1 → 116
110	Where are you living the rest of the time?	In another household in Umlalazi Municipa 1<br>In another municipality within Kwazulu-Natal . . . . 2<br>In a province other than Kwazulu-Natal in South Africa . . . . 3<br>In a country other than South Africa . 4	1, 2, 3 → 114
111	Which country is that?	Mozambique . . . . 1<br>Zimbabwe . . . . 2<br>Swaziland . . . . 3<br>OTHER ______________ 98<br>(SPECIFY)	1, 2, 3, 98 → 114
112	Where is the household you belong to located?	In Umlalazi Municipality . . . . 1<br>In another municipality within Kwazulu-Natal . . . . 2<br>In a province other than Kwazulu-Natal in South Africa . . . . 3<br>In a country other than South Africa . 4	1, 2, 3 → 114

NO.	QUESTIONS AND FILTERS	CODING CATEGORIES	SKIP
113	Which country is that?	Mozambique . . . . 1<br>Zimbabwe . . . . 2<br>Swaziland . . . . 3<br>OTHER ____________________ 98<br>(SPECIFY)	
114	How often do you come to visit this household?	Less than once a year . . . . 1<br>1-2 times a year . . . . 2<br>More than 2 times a year but less than once a month . . . . 3<br>At least once a month 4	
115	In total, in the last 12 months, how many days, weeks or months have you been living in the household where we are?	Number days . . . . [ ][ ]<br>Number weeks . . . . [ ][ ]<br>Number months . . . . [ ][ ]	
116	Have you changed your place of residence within the last 10 years?	YES . . . . 1<br>NO 2	 → 121
117	I'd like to know when you last moved.<br>Which month and year did you arrive in the place where you are currently living?	MONTH . . . . [ ][ ]<br>DON'T KNOW MONTH . . . . 99<br>YEAR . . . . [ ][ ][ ][ ]<br>DON'T KNOW YEAR 9999	
118	Where were you living before you moved the last time?	In this household . . . . 1<br>In another household in Umlalazi Municipality . . . . 2<br>In another municipality within Kwazulu-Natal . . . . 3<br>In a province other than Kwazulu-Natal in South Africa . . . . 4<br>In a country other than South Africa . 5	→ 120 (1–4)
119	Which country was that?	Mozambique . . . . 1<br>Zimbabwe . . . . 2<br>Swaziland . . . . 3<br>OTHER ____________________ 98<br>(SPECIFY)	
120	Why did you move the last time?	Work related . . . . 1<br>Family . . . . 2<br>Friends . . . . 3<br>OTHER ____________________ 98<br>(SPECIFY)	

NO.	QUESTIONS AND FILTERS	CODING CATEGORIES		SKIP
121	Are you currently married or living together with a man as if married?	YES, CURRENTLY MARRIED . . . .<br>YES, LIVING WITH A MAN . . . .<br>NO, NOT IN UNION	1<br>2<br>3	1, 2 → 124
122	Have you ever been married or lived together with a man as if married?	YES, FORMERLY MARRIED . . . .<br>YES, LIVED WITH A MAN . . . .<br>NO	1<br>2<br>3	3 → 124
123	What is your marital status now: are you widowed, divorced, or separated?	WIDOWED . . . .<br>DIVORCED . . . .<br>SEPARATED	1<br>2<br>3	
124	What kind of work do you do most of the time?	FARMER, FORESTRY . . . .<br>FISHING . . . .<br>SOLDIER,POLICEMAN . . . .<br>SALES, SERVICE WORKER . . . .<br>FACTORY WORKER . . . .<br>CLERICAL . . . .<br>PROFESSIONAL/MANAGER . . . .<br>(INCLUDES NURSE, TEACHER)<br>STUDENT . . . .<br>HOUSEWIFE . . . .<br>CONSTRUCTION . . . .<br>CLEANING / MAID . . . .<br>NONE . . . .<br><br>OTHER ______________________<br>(SPECIFY)	01<br>02<br>03<br>04<br>05<br>06<br>07<br><br>08<br>09<br>10<br>11<br>12<br><br>98	

SECTION 2. REPRODUCTION

NO.	QUESTIONS AND FILTERS	CODING CATEGORIES	SKIP
201	Now I would like to ask about all the births you have had during your life. Have you ever given birth?	YES . . . . 1<br>NO 2	 → 206
202	Do you have any sons or daughters to whom you have given birth who are now living with you?	YES . . . . 1<br>NO 2	 → 204
203	How many sons live with you?<br>And how many daughters live with you?<br>IF NONE, RECORD '00'.	SONS AT HOME . . . . [ ][ ]<br>DAUGHTERS AT HOME . . . . [ ][ ]	
204	Do you have any sons or daughters to whom you have given birth who are alive but do not live with you?	YES . . . . 1<br>NO 2	 → 206
205	How many sons are alive but do not live with you?<br>And how many daughters are alive but do not live with you?<br>IF NONE, RECORD '00'.	SONS ELSEWHERE . . . . [ ][ ]<br>DAUGHTERS ELSEWHERE [ ][ ]	
206	Have you ever given birth to a boy or girl who was born alive but later died?<br>IF NO, PROBE: Any baby who cried or showed signs of life but did not survive?	YES . . . . 1<br>NO 2	 → 208
207	How many boys have died?<br>And how many girls have died?<br>IF NONE, RECORD '00'.	BOYS DEAD . . . . [ ][ ]<br>GIRLS DEAD . . . . [ ][ ]	
208	SUM ANSWERS TO 203, 205, AND 207, AND ENTER TOTAL.<br>IF NONE, RECORD '00'.	TOTAL BIRTHS . . . . [ ][ ]	
209	CHECK 208:<br>Just to make sure that I have this right: you have had in TOTAL _____ births during your life. Is that correct?<br>YES [ ] ↓ NO [ ] → PROBE AND CORRECT 201-208 AS NECESSARY.	**IF '0' BIRTH**	→ **211**
210	In what month and year was your last child born?	MONTHS [ ][ ]<br>YEAR [ ][ ][ ][ ]<br>DON'T KNOW . 9999	
211	Are you currently pregnant?<br>IF YES, PROBE: How many months pregnant are you?	YES . . . . 1<br>NO . . . . 2<br>DK . . . . 9<br>MONTHS . . . . [ ]	

SECTION 3. PREGNANCY

NO.	QUESTIONS AND FILTERS	CODING CATEGORIES	SKIP
301	CHECK 210:<br>ONE OR MORE BIRTHS IN 2008 OR LATER ☐ ↓<br>NO BIRTHS IN 2008 OR LATER ☐ → 401		401
302	Now I would like to ask some questions about your LAST children born in the last five years.<br>What name was given to your last baby?	NAME __________	
303	Is (NAME) still alive?	YES . . . . 1<br>NO 2	
304	Did you see anyone for antenatal care for this pregnancy?	YES . . . . 1<br>NO 2	2 → 309
305	Whom did you see?<br>Anyone else?<br>PROBE TO IDENTIFY EACH TYPE OF PERSON AND RECORD ALL MENTIONED.	YES / NO / DK<br>*HEALTH PERSONNEL*<br>Doctor . . . . . 1 2 9<br>Nurse/Midwife 1 2 9<br>Auxiliary midwife 1 2 9<br>*OTHER PERSON*<br>Traditional birth attendant ... 1 2 9<br>Community Health Worker 1 2 9<br>OTHER 1 2 9<br>__________ (SPECIFY)	
306	Where did you receive antenatal care for this pregnancy?<br>Anywhere else?<br>PROBE TO IDENTIFY EACH TYPE OF SOURCE.<br>IF UNABLE TO DETERMINE IF PUBLIC OR PRIVATE SECTOR, WRITE THE NAME OF THE PLACE.<br>__________ (NAME OF PLACE(S))	YES / NO / DK<br>*HOME*<br>YOUR HOME . 1 2 9<br>OTHER HOME . 1 2 9<br>*PUBLIC SECTOR*<br>GOVT. HOSPITAL 1 2 9<br>GOVT. CLINIC 1 2 9<br>GOVT. MOBILE CLINIC . . . . . 1 2 9<br>OTHER PUBLIC SECTOR 1 2 9<br>__________ (SPECIFY)<br>*PRIVATE MED. SECTOR*<br>Pvt. Hospital/ Clinic 1 2 9<br>Other private sector 1 2 9<br>__________ (SPECIFY)<br>OTHER 1 2 9<br>__________ (SPECIFY)	
307	How many months pregnant were you when you first received antenatal care for this pregnancy?	MONTHS . . . |__|__| DON'T KNOW . . . 99	

NO.	QUESTIONS AND FILTERS	CODING CATEGORIES	SKIP
308	How many times did you receive antenatal care during this pregnancy?	NUMBER OF TIMES [ ][ ]<br>DON'T KNOW . . . 99	
309	Who assisted with the delivery of (NAME)?<br><br>Anyone else?<br><br>PROBE FOR THE TYPE(S) OF PERSON(S) AND RECORD ALL MENTIONED.<br><br>IF RESPONDENT SAYS NO ONE ASSISTED, PROBE TO DETERMINE WHETHER ANY ADULTS WERE PRESENT AT THE DELIVERY.	YES / NO / DK<br>*HEALTH PERSONNEL*<br>Doctor . . . . . 1 2 9<br>Nurse/Midwife 1 2 9<br>Auxiliary midwife 1 2 9<br><br>*OTHER PERSON*<br>Traditional birth attendant ... 1 2 9<br>Relative / Friend 1 2 9<br>OTHER 1 2 9<br>________ (SPECIFY)<br>NO ONE ASSISTED 1 2 9	
310	Where did you give birth to (NAME)?<br><br>PROBE TO IDENTIFY THE TYPE OF SOURCE.<br><br>IF UNABLE TO DETERMINE IF PUBLIC OR PRIVATE SECTOR, WRITE THE NAME OF THE PLACE.<br><br>________________<br>(NAME OF PLACE)	HOME<br>YOUR HOME . 31<br>OTHER HOME . 32<br>PUBLIC SECTOR<br>GOVT. HOSPITAL 11<br>GOVT. CLINIC 12<br>GOVT. MOBILE CLINIC 13<br>OTHER PUBLIC SECTOR<br>________ 18<br>(SPECIFY)<br>PRIVATE MED. SECTOR<br>PVT. HOSPITAL/ CLINIC 21<br>OTHER PRIVATE MED. SECTOR<br>________ 28<br>(SPECIFY)<br>OTHER________ 98<br>(SPECIFY)	31 → 312<br><br>98 → 312
311	How long after (NAME) was delivered did you stay there?<br><br>IF LESS THAN ONE DAY, RECORD HOURS.<br>IF LESS THAN ONE WEEK, RECORD DAYS.	HOURS 1 [ ][ ]<br>DAYS 2 [ ][ ]<br>WEEKS 3 [ ][ ]<br>DON'T KNOW . . . 99	
312	Did you ever breastfeed (NAME)?	YES . . . . 1<br>NO 2	2 → 401
313	CHECK 303:<br><br>IS (NAME) LIVING?	LIVING [ ] ↓ DEAD [ ] →	DEAD → 401
314	Are you still breastfeeding (NAME)?	YES . . . . 1<br>NO 2	1 → 401
315	What age was (NAME) when you stopped to breastfeed him/her?	MONTHS . . . [ ][ ]<br>DON'T KNOW . . . 99	

4. HIV/AIDS

NO.	QUESTIONS AND FILTERS	CODING CATEGORIES	SKIP
401	Now I would like to talk about something else. Have you ever heard of an illness called HIV / AIDS?	YES . . . . . 1<br>NO 2	 → 420
402	Do you think circumcision can prevent the transmission of HIV / AIDS?	YES . . . . . 1<br>NO . . . . . 2<br>DON'T KNOW 9	
403	Can HIV / AIDS be transmitted from a mother to her baby?	YES NO DK<br>IN GENERAL 1 2 9	If No or DK<br>→ 405
	If yes, Can HIV /AIDS be transmitted,<br>During pregnancy?<br>During delivery?<br>By breastfeeding?	 DURING PREG. . . . . . 1 2 9<br>DURING DELIVERY . . . 1 2 9<br>BREASTFEEDING . . . 1 2 9	
404	Are there any special drugs that a doctor or a nurse can give to a woman infected with the HIV/AIDS virus to reduce the risk of transmission to the baby?	YES . . . . . 1<br>NO . . . . . 2<br>DON'T KNOW 9	
405	Do you know of a place where people can go to get tested for HIV?	YES . . . . . 1<br>NO 2	 → 407
406	Where is that place?<br><br>Any other place?<br><br>PROBE TO IDENTIFY EACH TYPE OF SOURCE.<br><br>IF UNABLE TO DETERMINE IF PUBLIC OR PRIVATE SECTOR, WRITE THE NAME OF THE PLACE.<br><br>________________<br>(NAME OF PLACE(S))	YES NO DK<br>*PUBLIC SECTOR*<br>Govt. Hospital . . . . 1 2 9<br>Govt. Clinic . . . . 1 2 9<br>Govt. Stand-alone VCT Ce 1 2 9<br>Govt. Family Planning Clir 1 2 9<br>Govt. Mobile Clinic . . . 1 2 9<br>Govt. Home by CCG . 1 2 9<br>Govt. School based Clinic. 1 2 9<br>OTHER Public Sector . 1 2 9<br>__________<br>(SPECIFY)<br>*PRIVATE MEDICAL SECTOR*<br>Pvt. Hospital / Clinic / Dr 1 2 9<br>Pharmacy . . . . 1 2 9<br>OTHER Private Sector . 1 2 9<br>__________<br>(SPECIFY)<br>*MSF*<br>MSF Fixed testing site . 1 2 9<br>MSF Mobile sites (M1SS) 1 2 9<br>MSF CHA (at home) . . . 1 2 9<br>OTHER MSF . . . . 1 2 9<br>__________<br>(SPECIFY)<br>OTHER 1 2 9<br>__________<br>(SPECIFY)	
407	Now I would like to ask you some questions about your own experience of HIV testing. Your answers are completely private. This form will not have your name anywhere on it; instead you will only be identified by a number. Have you had an HIV test before?	YES . . . . . 1<br>NO 2	 → 420
408	How many times have you had an HIV test in your lifetime?	NUMBER OF TIMES . . . . [ ][ ]<br><br>DON'T KNOW 99	

NO.	QUESTIONS AND FILTERS	CODING CATEGORIES	SKIP
409	In which month and year was your most recent test?	MONTH . . . . [ ][ ]<br>DON'T KNOW . . . . 99<br>YEAR . . . . [ ][ ][ ][ ]<br>DON'T KNOW 9999	
410	Where was the test done?<br><br>PROBE TO IDENTIFY THE TYPE OF SOURCE.<br><br>IF UNABLE TO DETERMINE IF PUBLIC OR PRIVATE SECTOR, WRITE THE NAME OF THE PLACE.<br><br>______________________<br>(NAME OF PLACE)	*PUBLIC SECTOR*<br>Govt. Hospital . . . . 11<br>Govt. Clinic . . . . 12<br>Govt. Stand-alone VCT Center . . . 13<br>Govt. Family Planning Clinic. . . . . 14<br>Govt. Mobile Clinic . . . . 15<br>Govt. Home by CCG . . . . 16<br>Govt. School based Clinic. . . . . 17<br>OTHER Public Sector . . . . 18<br>______________<br>(SPECIFY)<br>*PRIVATE MEDICAL SECTOR*<br>Pvt. Hospital / Clinic / Dr . . . . 21<br>Pharmacy . . . . 22<br>OTHER Private Sector . . . . 28<br>______________<br>(SPECIFY)<br>*MSF*<br>MSF Fixed testing site . . . . 31<br>MSF Mobile sites (M1SS) . . . . 32<br>MSF CHA (at home) . . . . 33<br>OTHER MSF . . . . 38<br>______________<br>(SPECIFY)<br>OTHER ______________ 98<br>(SPECIFY)	
411	Did you get the results of the test?	YES . . . . 1<br>NO 2	 → 413
412	I would like to ask you the result of your latest HIV test, but I want to remind you again that you should only answer the question if you feel comfortable. If you feel comfortable, could you tell me the result of your latest HIV test?	POSITIVE . . . . 1<br>NEGATIVE . . . . 2<br>INDETERMINATE . . . . 3<br>REFUSE TO ANSWER . . . . 4<br>DON'T KNOW 9	
413	How high do you consider/estimate your own risk of contracting HIV / AIDS?	No risk . . . . 1<br>Low . . . . 2<br>Moderate . . . . 3<br>High . . . . 4<br>Already know I'm HIV positive . . . . 5<br>DON'T KNOW 9	

NO.	QUESTIONS AND FILTERS	CODING CATEGORIES	SKIP
414	CHECK 208: BIRTH(S) [ ] ↓	NO BIRTHS [ ]	→ 420
415	CHECK 210<br>LAST BIRTH DURING OR AFTER 2011 [ ] ↓	LAST BIRTH BEFORE 2011 [ ]	→ 420
416	CHECK 304 FOR LAST BIRTH:<br>HAD ANTENATAL CARE [ ] ↓	NO ANTENATAL CARE [ ]	→ 420
417	Were you tested for HIV as part of your antenatal care?	YES . . . 1<br>NO . . . 2	 → 420
418	How many months pregnant were you when you were first had an HIV test as a part of your antenatal care for this pregnancy?	MONTHS . . . [ ][ ]<br>DON'T KNOW . . . 99	
419	Did you get the results of the test?	YES . . . 1<br>NO . . . 2	
420	Now I would like to ask you some questions about your recent sexual activity. Let me assure you again that your answers are completely confidential and will not be told to anyone. If we come to any question that you don't want to answer, just let me know and we will go to the next question.<br><br>When was the last time you had sexual intercourse?	Never had sexual intercourse . . . 1<br>Within the last 30 days . . . 2<br>More than 30 days ago but less 1 year 3<br>More than 12 months ago . . . 4	→ 437<br>→ 421<br>→ 421<br>→ 437
421	We would like to ask you some questions about your last partners<br>Please think about the last person you had sexual intercourse with<br><br>When was the <u>last time</u> you had sexual intercourse with this person?	MONTH . . . [ ][ ]<br>DON'T KNOW . . . 99<br>YEAR . . . [ ][ ][ ][ ]	
422	Did you use a condom the <u>last time</u> you had sexual intercourse with this person?	YES . . . 1<br>NO . . . 2	
423	How often did you use condoms when you had sexual intercourse with this person?	ALWAYS . . . 1<br>SOMETIMES . . . 2<br>NEVER . . . 3	
424	When was the <u>first time</u> you had sexual intercourse with this person?	MONTH . . . [ ][ ]<br>DON'T KNOW . . . 99<br>YEAR . . . [ ][ ][ ][ ]<br>DON'T KNOW . . . 9999	
425	What was your relationship to this person with whom you had sexual intercourse?<br><br>*Transactional partner is defined as sex in exchange for money, gifts, good grades or other favours*	Wife/Husband . . . 1<br>Live-in partner . . . 2<br>Girlfriend/Boyfriend . . . 3<br>Casual acquaintance . . . 4<br>Transactional partner . . . 5<br><br>OTHER ______ 98<br>(SPECIFY)	
426	Apart from this person, have you had sexual intercourse with any other person in the last 12 months?	YES . . . 1<br>NO . . . 2	 → 436

NO.	QUESTIONS AND FILTERS	CODING CATEGORIES	SKIP
427	When was the last time you had sexual intercourse with this person?	MONTH .... ☐☐<br>DON'T KNOW .... 99<br>YEAR .... ☐☐☐☐	
428	Did you use a condom the last time you had sexual intercourse with this person?	YES .... 1<br>NO 2	
429	When was the first time you had sexual intercourse with this person?	MONTH .... ☐☐<br>DON'T KNOW .... 99<br>YEAR .... ☐☐☐☐<br>DON'T KNOW 9999	
430	What was your relationship to this second person with whom you had sexual intercourse?<br><br>*Transactional partner is defined as sex in exchange for money, gifts, good grades or other favours*	Wife/Husband .... 1<br>Live-in partner .... 2<br>Girlfriend/Boyfriend........................ 3<br>Casual acquaintance .... 4<br>Transactional partner .... 5<br><br>OTHER ______________ 98<br>(SPECIFY)	
431	Apart from this person, have you had sexual intercourse with any other person in the last 12 months?	YES .... 1<br>NO 2	 → 436
432	When was the last time you had sexual intercourse with this person?	MONTH .... ☐☐<br>DON'T KNOW .... 99<br>YEAR .... ☐☐☐☐	
433	Did you use a condom the last time you had sexual intercourse with this person?	YES .... 1<br>NO 2	
434	When was the first time you had sexual intercourse with this person?	MONTH .... ☐☐<br>DON'T KNOW .... 99<br>YEAR .... ☐☐☐☐<br>DON'T KNOW 9999	
435	What was your relationship to this person with whom you had sexual intercourse?<br><br>*Transactional partner is defined as sex in exchange for money, gifts, good grades or other favours*	Wife/Husband .... 1<br>Live-in partner .... 2<br>Girlfriend/Boyfriend .... 3<br>Casual acquaintance .... 4<br>Transactional partner .... 5<br><br>OTHER ______________ 98<br>(SPECIFY)	
436	In total, with how many different partners have you had sexual intercourse within the last 12 months?	Number of partners .... ☐☐<br><br>Don't know 99	
437	THANK THE PATIENT FOR HER PARTICIPATION<br><br>START PRE-COUNSELLING		

SECTION 5. ART Coverage

NO.	QUESTIONS AND FILTERS	CODING CATEGORIES	SKIP
	We are now going to talk about your knowledge of care and treatment of HIV / AIDS. In order for MSF to improve the services provided, it is very important to answer in the truest way possible. There will be no judgement and no consequences.		
501	Have you already had a HIV test that showed you were infected with HIV/AIDS?	YES . . . . 1<br>NO 2	
502	CHECK (CROSS CHECK WITH 412)<br>KNEW HIV STATUS ☐ ↓	NEW PATIENT ☐	→ END
	We are now going to talk about care and treatment of HIV / AIDS. We know they are many reasons which could lead the patient to withdraw from HIV care. Again it is very important to answer in the truest way possible, there will be no judgement and no consequences		
503	When were you first tested positive for HIV / AIDS?	MONTH . . . . [ ][ ]<br>DON'T KNOW . . . . 99<br>YEAR . . . . [ ][ ][ ][ ]<br>DON'T KNOW 9999	
504	Where was this test done?<br><br>PROBE TO IDENTIFY THE TYPE OF SOURCE.<br><br>IF UNABLE TO DETERMINE IF PUBLIC OR PRIVATE SECTOR, WRITE THE NAME OF THE PLACE.<br><br>__________________<br>(NAME OF PLACE)	*PUBLIC SECTOR*<br>Govt. Hospital ……………………. 11<br>Govt. Clinic ………………………. 12<br>Govt. Stand-alone VCT Center 13<br>Govt. Family Planning Clinic. . . . . 14<br>Govt. Mobile Clinic …… 15<br>Govt. Home by CCG … 16<br>Govt. School based Clinic. . . . . . . 17<br>OTHER Public Sector 18<br>__________<br>(SPECIFY)<br>*PRIVATE MEDICAL SECTOR*<br>Pvt. Hospital / Clinic / Dr …………. 21<br>Pharmacy …….. 22<br>OTHER Private Sector ………… 28<br>__________<br>(SPECIFY)<br>*MSF*<br>MSF Fixed testing site ………………. 31<br>MSF Mobile sites (M1SS) …… 32<br>MSF CHA (at home) … 33<br>OTHER MSF 38<br>__________<br>(SPECIFY)<br>OTHER __________ 98<br>(SPECIFY)	

NO.	QUESTIONS AND FILTERS	CODING CATEGORIES	SKIP
505	After you discovered you were infected with the virus that causes HIV / AIDS, did you ever seek care for the HIV / AIDS infection?	YES . . . . . 1<br>NO 2	 2 → END
506	Did you have any blood samples taken to check your CD4 count when you first went to receive care?	YES . . . . . 1<br>NO . . . . . 2<br>DON'T KNOW 9	 2, 9 → 508
507	Did you get the results of the CD4 blood test?	YES . . . . . 1<br>NO . . . . . 2<br>DON'T KNOW 9	
508	Have you ever initiated ART, antiretroviral treatment drugs against HIV/ AIDS?	YES . . . . . 1<br>NO 2	 2 → 516
509	When did you first start Antiretroviral therapy?<br><br>(IF NEEDED CHECK ON THE HEALTH BOOKLET)	MONTH . . . . . [ ][ ]<br>DON'T KNOW . . . . . 99<br>YEAR . . . . . [ ][ ][ ][ ]<br>DON'T KNOW 9999	
510	Are you still receiving ART, antiretroviral treatment drugs against HIV / AIDS?	YES . . . . . 1<br>NO 2	 2 → 522
511	When was your last consultation?<br><br>(IF NEEDED CHECK ON THE HEALTH BOOKLET)	MONTH . . . . . [ ][ ]<br>DON'T KNOW . . . . . 99<br>YEAR . . . . . [ ][ ][ ][ ]<br>DON'T KNOW 9999	
512	Where are you receiving ART?<br><br>PROBE TO IDENTIFY THE TYPE OF SOURCE.<br><br>IF UNABLE TO DETERMINE IF PUBLIC OR PRIVATE SECTOR, WRITE THE NAME OF THE PLACE.<br><br>________________<br>(NAME OF PLACE)	*PUBLIC SECTOR*<br>Govt. HOSPITAL 11<br>Govt. CLINIC 12<br>Govt. MOBILE CLINIC 13<br>OTHER Public Sector 18<br>__________<br>(SPECIFY)<br><br>*PRIVATE MEDICAL SECTOR*<br>Pvt. Hospital / Clinic / Dr 21<br>OTHER Private Sector 28<br>__________<br>(SPECIFY)<br><br>*COMMUNITY GROUPS*<br>Clubs or<br>Community ART Groups (CAG) … 41<br><br>OTHER SOURCE 98<br>__________<br>(SPECIFY)	

NO.	QUESTIONS AND FILTERS	CODING CATEGORIES	SKIP
513	Where is this place located?	In Umlalazi Municipality .... 1<br>In another municipality within<br>Kwazulu-Natal .... 2<br>In a province other than Kwazulu-Natal<br>in South Africa .... 3<br>In a country other than South Africa ... 4	1 → 515<br>2, 3 → END
514	Which country is that?	Mozambique .... 1<br>Zimbabwe .... 2<br>Swaziland .... 3<br>Other .... 98<br>___ (SPECIFY)	→ END
515	What is the name of this place?<br><br>SEE CODES OF FACILITIES IN ANNEX	___ (SPECIFY) |__|__|	→ END
516	Are you still followed up for the HIV / AIDS infection?	YES .... 1<br>NO 2	2 → 522
517	When was your last consultation?<br><br>(IF NEEDED CHECK ON THE HEALTH BOOKLET)	MONTH .... |__|__| DON'T KNOW .... 99<br>YEAR .... |__|__|__|__| DON'T KNOW 9999	
518	Where are you now receiving care?<br><br>PROBE TO IDENTIFY THE TYPE OF SOURCE.<br><br>IF UNABLE TO DETERMINE IF PUBLIC OR PRIVATE SECTOR, WRITE THE NAME OF THE PLACE.<br><br>___ (NAME OF PLACE)	*PUBLIC SECTOR*<br>Govt. HOSPITAL .... 11<br>Govt. CLINIC .... 12<br>Govt. MOBILE CLINIC .... 13<br>OTHER Public Sector ... 18<br>___ (SPECIFY)<br><br>*PRIVATE MEDICAL SECTOR*<br>Pvt. Hospital / Clinic / Dr .... 21<br>OTHER Private Sector ... 28<br>___ (SPECIFY)<br>OTHER SOURCE 98<br>___ (SPECIFY)	
519	Where is this place located?	In Umlalazi Municipality 1<br>In another municipality within<br>Kwazulu-Natal .... 2<br>In a province other than Kwazulu-Natal<br>in South Africa .... 3<br>In a country other than South Africa ... 4	1 → 521<br>2, 3 → END
520	Which country is that?	Mozambique .... 1<br>Zimbabwe .... 2<br>Swaziland .... 3<br>Other .... 98<br>___ (SPECIFY)	→ END
521	What is the name of this place?<br><br>SEE CODES OF FACILITIES IN ANNEX	___ (SPECIFY) |__|__|	→ END
522	Why did you stop?	NO ONE WAS ATTENDING 1<br>NO DRUGS AVAILABLE 2<br>UNFRIENDLY STAFF 3<br>SPONTANEOUS (NO SPECIFIC REASOI 4<br>ADVISED TO STOP 5<br>THOUGHT I WAS CURED/ FEEL GOOD 6<br>SIDE EFFECTS 7<br>MOVED AWAY 8<br>TRANSPORT COSTS 9<br>STOP PMTCT 10<br><br>OTHER ___ (SPECIFY) 98	

INTERVIEWER'S OBSERVATIONS

TO BE FILLED IN AFTER COMPLETING INTERVIEW

COMMENTS ABOUT RESPONDENT:

COMMENTS ON SPECIFIC QUESTIONS:

ANY OTHER COMMENTS:

SUPERVISOR'S OBSERVATIONS

NAME OF SUPERVISOR: ______________________ DATE: ______________________

EDITOR'S OBSERVATIONS

NAME OF EDITOR: ______________________ DATE: ______________________
